# Transcriptome analysis of *Enterococcus faecalis* in response to alkaline stress

**DOI:** 10.3389/fmicb.2015.00795

**Published:** 2015-08-07

**Authors:** Shujun Ran, Bin Liu, Wei Jiang, Zhe Sun, Jingping Liang

**Affiliations:** Shanghai Key Laboratory of Stomatology, Department of Endodontics and Operative Dentistry, School of Medicine, Ninth People's Hospital, Shanghai Jiao Tong UniversityShanghai, China

**Keywords:** *E. faecali*s, alkaline stress, biofilms, transcriptome, genome, sequencing

## Abstract

*Enterococcus faecalis* is the most commonly isolated species from endodontic failure root canals; its persistence in treated root canals has been attributed to its ability to resist high pH stress. The goal of this study was to characterize the *E. faecalis* transcriptome and to identify candidate genes for response and resistance to alkaline stress using Illumina HiSeq 2000 sequencing. We found that *E. faecalis* could survive and form biofilms in a pH 10 environment and that alkaline stress had a great impact on the transcription of many genes in the *E. faecalis* genome. The transcriptome sequencing results revealed that 613 genes were differentially expressed (DEGs) for *E. faecalis* grown in pH 10 medium; 211 genes were found to be differentially up-regulated and 402 genes differentially down-regulated. Many of the down-regulated genes found are involved in cell energy production and metabolism and carbohydrate and amino acid metabolism, and the up-regulated genes are mostly related to nucleotide transport and metabolism. The results presented here reveal that cultivation of *E. faecalis* in alkaline stress has a profound impact on its transcriptome. The observed regulation of genes and pathways revealed that *E. faecali*s reduced its carbohydrate and amino acid metabolism and increased nucleotide synthesis to adapt and grow in alkaline stress. A number of the regulated genes may be useful candidates for the development of new therapeutic approaches for the treatment of *E. faecalis* infections.

## Introduction

*Enterococcus* spp. are natural inhabitants of the gastrointestinal tracts of humans and animals (Creti et al., 2004), but they can also be found in water, soil, and vegetables (Burgos et al., 2009). The two most important species, *Enterococcus faecium* and *Enterococcus faecalis*, are most frequently implicated in human and animal infections (Aakra et al., 2005). They have been reported to be associated with oral mucosal lesions in immunocompromised patients, periodontitis, and root canal infections (Wahlin and Holm, 1988; Rams et al., 1992; Siqueira and Rôças, 2004).

*E. faecalis* is the species that is most commonly isolated from the root canals of teeth with failed endodontic treatment (Sundqvist et al., 1998). Moreover, *E. faecalis* was most often detected as the single species in persistently infected root canals. It has been reported that in teeth with necrotic pulps, *E. faecalis* has rarely been isolated by culturing (4%), but has frequently been detected by PCR (82%); in fact, it is detected as occurring frequently in teeth with failing endodontic treatment. *E. faecalis* may occur in low numbers in necrotic pulp, keeping it from being cultured from these samples (Gomes et al., 2006). Moreover, the success rate for the teeth from which *E. faecalis* was isolated after removal of the earlier root filling was somewhat lower (66%) than the overall success rate of re-treatment (74%) (Sundqvist et al., 1998). Therefore, *E. faecalis* involved in the etiology of endodontic failure may be attributed to the organism being present in necrotic pulps that survived until chemo-mechanical procedures were performed and intracanal medications were given, which caused ecological changes in the root canals (Love, 2001; Gomes et al., 2003).

Calcium hydroxide is known to be one of the most effective and commonly used endodontic medicaments because of the bactericidal effects derived from its strong alkaline properties; it can provide an extreme alkalinity of approximately pH 12 (Athanassiadis et al., 2007). However, some studies report that calcium hydroxide is not so effective against *E. faecalis* (Evans et al., 2002; Gomes et al., 2003). The persistence of *E. faecalis* in treated root canals has been attributed to its ability to resist the high pH of the antimicrobial agents commonly used during treatment (Waltimo et al., 2005). It has even been shown that *E. faecalis* can directly form biofilms on calcium hydroxide paste in medicated root canals (Distel et al., 2002), and a slight pH increase enhances *E. faecalis* adhesion to collagen type I (the major organic component of dentine) (Kayaoglu et al., 2005). Moreover, an extreme pH cannot be achieved within dentinal tubules because of the buffering effects of dentin (Haapasalo et al., 2000).

The antimicrobial activity of Ca(OH)_2_ relates to its release of hydroxide ions (Freeman and Crapo, 1982). The destruction of phospholipids, the structural components of the cellular membrane, can be caused by the induction of lipid peroxidation by hydroxide ions (Halliwell, 1987). Genes are destroyed when hydroxide ions react with bacterial DNA and cause the denaturation of the strands (Imlay and Linn, 1988). It has been reported that the expression of some stress response genes are increased markedly in alkaline media (Appelbe and Sedgley, 2007). An analysis of stress proteins revealed that 37 polypeptides were amplified when subjected to alkaline shock (Flahaut et al., 1997). Although a number of virulence-related traits have been established, no comprehensive genomic or transcriptomic studies have been conducted to investigate how *E. faecalis* resists alkaline stress. To identify the potential genetic traits or gene regulatory features that allow *E. faecalis* to survive in alkaline stress, we performed genomic analysis and investigated the growth capacity and transcriptome profiling in pH 10 media, which may ultimately contribute to the development of strategies for the prevention of *E. faecalis* infections and improve root canal treatment strategies.

## Materials and methods

### Bacterial strains and growth conditions

*E. faecalis* (ATCC 33186) was used in all experiments and stored in a 50% (v/v) glycerol solution at −80°C. Bacteria were prepared for all experiments by the inoculation of tryptic soy broth (TSB) containing 1.7% tryptone, 0.3% polypeptone, 0.1% yeast extract (Sangon, Shanghai, China), 0.5% NaCl, 0.25% glucose, and 0.25% dipotassium phosphate, which was incubated aerobically at 37°C for 16–18 h. Under these conditions, the cultures entered stationary phase at an optical density of 1.1 at 600 nm (OD_600 nm_). The alkaline media (pH 10) was adjusted with maleic acid and K_2_CO_3_(Kakinuma and Igarashi, 1990) and sterilized through a 0.22-μm (pore size) filter unit (Millipore, Millex-GP, USA).

### Biofilm assay

Confocal microscopy was performed on *E. faecalis* biofilms grown on glass coverslips. Sterile glass coverslips that were placed on the bottom of six-well culture plates and submerged with 6 mL of alkaline and TSB medium were seeded with a 1:100 dilution of *E. faecalis* from an overnight culture (OD_600 nm_ = 1.1) and grown for 24 h at 37°C. Just prior to imaging, the biofilms were gently rinsed two times with sterile phosphate-buffered saline, followed by 15 min of staining with 100 μl of the fluorescent stains SYTO9 and PI (Invitrogen, California, USA), which were diluted in PBS in proportions of 1.5:1.5:1000. The slides were visualized by an inverted Leica TCS SP2 laser scanning confocal microscope (Leica Microsystems, Mannheim, Germany). A 488/543-nm double dichroic mirror was used as an excitation beam splitter, and a 405-nm short-pass filter divided green and red fluorescence between the photomultipliers. Identification of viable cells and biofilm quantification was carried out using the Leica confocal software.

### Genome sequencing

Bacteria were incubated aerobically in TSB at 37°C for 24 h. Cells (30 ml) were collected by centrifugation (4000 rpm, 15 min) at 4°C, and the pellets were immediately frozen in liquid nitrogen and kept at −80°C prior to DNA extraction. Genomic DNA extraction was carried out with a bacterial genomic DNA purification kit (EdgeBio, Maryland, USA) according to the manufacturer's instructions. The genome library was constructed by DNA fragmentation with Covaris; both ends of the DNA fragments were fixed with complementary primers to form a “bridge” for subsequent PCR amplification and the generation of single stranded DNA. Sequencing was carried out on an Illumina HiSeq 2000 platform (Majorbio Bio-Pharm Technology Co., Ltd., Shanghai, China).

### RNA extraction and sequencing

Biofilm cells were grown for 24-h in TSB OR in pH 10 medium and were harvested by centrifugation (4000 rpm, 15 min) at 4°C. Cells were then resuspended with TRIzol (Invitrogen, Carlsbad, USA) and disrupted with a Mini-Bead beater (Biospec, California, USA). Total RNA was isolated according to the manufacturer's instructions for TRIzol-chloroform extraction (Invitrogen, California, USA). Genomic DNA was removed with Turbo DNase. Ribosomal RNA (rRNA) was removed with a Ribo-Zero Magnetic kit (G+/G-Bacteria) (Epicentre, Wisconsin, USA). The cDNA library was constructed with extracted mRNA according to a TruseqTM RNA sample prep kit (Illumina, California, USA). The sample was purified and ligated to the genomic adapters provided by Illumina. The purified samples were amplified by 15-cycle PCR. After ligation, the sample was loaded on a 2% agarose E-gel (Invitrogen). The targeted fragments were excised from the gel and purified using Certified Low Range Ultra Agarose (Bio-Rad). The sample was quantified using TBS380 Picogreen (Invitrogen). Sequencing was carried out on the Illumina HiSeq 2000 platform (Majorbio Bio-Pharm Technology Co., Ltd., Shanghai, China).

### Data analysis

The raw image data were converted to sequence data through Base Calling. Then, the raw sequence data were filtered with SeqPrep and Sickle software to obtain clean data. The clean reads from each sample were mapped to the reference genome that was obtained from preceding genome sequencing by Bowtie software (Langmead et al., 2009). The predicted protein sequence from the open reading frames (ORFs) were annotated with NR, string, and a gene database using blastp. Based on their homology to protein families, proteins predicted for *E. faecalis* were assigned parental (i.e., level 2) Gene Ontology (GO) terms (http://www.geneontology.org/). Deduced proteins with homologs in other organisms were used to determine the Clusters of Orthologous Groups of proteins (COG) item; the COG functional categories were assigned with the STRING database (String v9.0). Based on the KEGG (Kyoto Encyclopedia of Genes and Genomes database), all of the genes were mapped to the KEGG gene database (GENES) by using the BLAST algorithm (blastx/blastp2.2.24+) to find the potential biological pathway of the genes (Camacho et al., 2009).

Differentially expressed genes (DEGs) were obtained based on the RPKM/FPKM [Reads/(Fragments) Per Kilobase of exon model per Million] followed by a multiple hypothesis testing, False Discovery Rate (FDR) control (Trapnell et al., 2010) to correct for the *p*-value. Genes with an FDR value ≤ 0.05 and |logFC| ≥ 1 were assigned as differentially expressed. Differences in gene expression were also analyzed by the R package, EdgeR (Robinson et al., 2010).

## Results

### Biofilm formation of *E. faecalis* by CLSM

**Figure 1:**
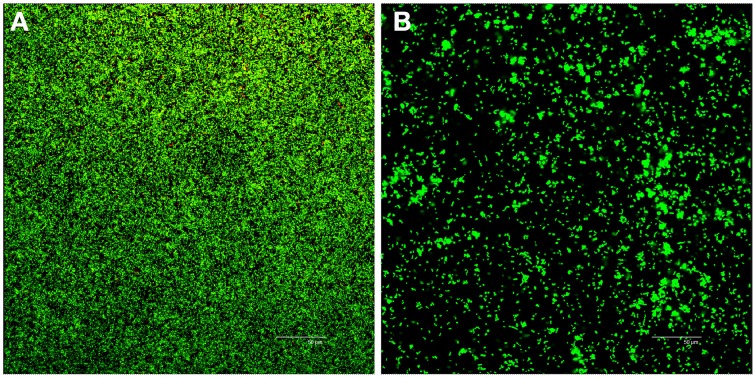
**Confocal analysis of 24-h biofilms of *E. faecalis* in TSB and pH 10 media**. Live bacteria are green, and dead cells are visualized in red. Panels **(A,B)** are representative biofilm projections of *E. faecalis* in control group (TSB) and pH 10 media, respectively.

Confocal laser scanning microscopy (CLSM) analysis of 24-h-old biofilms in the control group (pH 7.4) showed a dense and compact biofilm with a complex architecture (Figure 1A); in alkaline media (pH 10), CLSM showed poor biofilms that were mainly composed of isolated microcolonies with sparse cells on the glass surface (Figure 1B). The viable bacteria and biofilm biomass were significantly reduced in pH 10 media compared with the control group (Independent-samples *T*-test, *P* < 0.05).

### Genomic analysis

Because there is no sequence data publicly available for *E. faecalis* ATCC33186, genome sequencing was performed before transcriptional analysis to obtain a detailed account of the genetic expression and to validate the performance of our RNA-Seq. The genome sequence was determined by Shanghai Majorbio Bio-pharm Technology Co., Ltd. (Shang-hai, China), using Illumina HiSeq2000 paired-end sequencing technology (300-bp library). A total of 433,968,680 high-quality base pairs (4,339,345 high-quality reads, with an average read length of 91 bp), giving 148.7-fold coverage of the genome, were assembled into 73 contigs and 63 scaffolds using SOAPdenovo software (http://soap.genomics.org.cn/). The genome of *E. faecalis* ATCC33186 consists of 63 scaffolds of 2,918,140 bp and had an average G+C content of 37.36%. There are a total of 2824 putative open reading frames (with an average size of 896 bp) predicted by Glimmer 3.0. The detailed sequence information of the genome is listed in Table 1.

**Table 1:** **General information of the genome from *E. faecalis ATCC33186***.

Coding DNA Sequence (CDS)	2824
Genome length	2918140 bp
GC content in gene region (%)	38
Gene average length	896 bp
GC content in non-coding sequence (%)	33
N rate^*^	0%

* *N rate is the percentage of unknown bases. In the process of sequence assembly, the unknown bases between contigs were filled with N*.

Using the published DNA sequence of V583(NC_004668.1), OG1RF(NC_017316.1), and ATCC 29212(NZ_CP008816.1) as reference, *E. faecalis* ATCC33186 shares 2362, 2284, and 2402 ORFs, respectively, as well as the 3 rRNA genes and 41 tRNA genes (Table 2). Their genome size and GC content were similar (ranging from 2.7 to 3.2 Mb and from 37.4 to 37.8%, respectively).

**Table 2:** **Gene features of ATCC33186, compared to V583, OG1RF, and ATCC 29212**.

	**ATCC33186**	**V583**	**OG1RF**	**ATCC 29212**
Size (bp)	2,918,140	3,218,031	2,739,625	2,939,973
GC content (%)	37.4	37.5	37.8	37.5
Gene	2868	3257	2640	3053
rRNA	3^*^	12	12	12
tRNA	41^*^	68	58	61
ORFs	2824	3113	2559	2908
		**Compared to V583**	**Compared to OGIRF**	**Compared to ATCC 29212**
ORFs common to both strains		2362	2284	2402
Unique ORFs		462	540	422
Similar to known proteins		412	456	358
Conserved hypotheticals		46	79	60
No database match		4	5	4

* *The big difference in number of rRNA and tRNA compared with the reported strains partly attribute to the absence of completely assembled chromosome of strain ATCC33186 in the present study*.

### Gene ontology assignments

**Figure 2:**
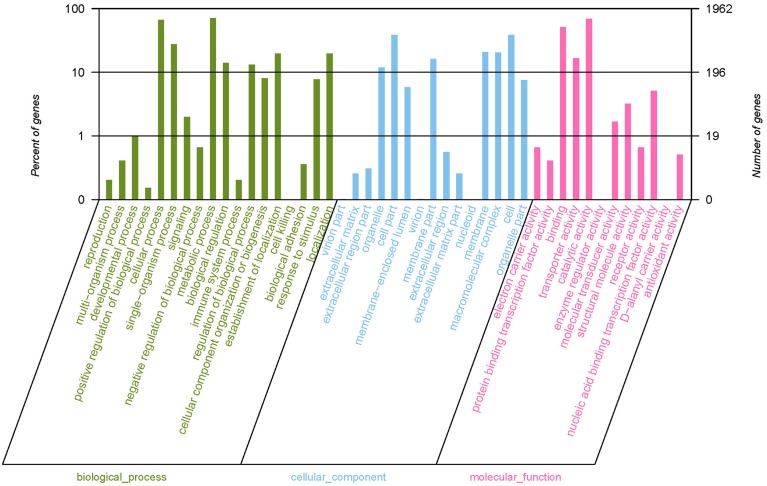
**Gene ontology (GO) terms for the transcriptome sequences of *E*. *faecalis***. Most of the annotated sequences can be divided into three major categories, including biological process **(A)**, cellular component **(B)**, and molecular function **(C)**. The vertical scale on the left indicated the the percentage of each sub-categories of GO terms to the total 1962 genes which were successfully annotated by GO assignments. The vertical scale on the right side indicated the number of each sub-categories of GO terms. Because some genes were assigned to more than one GO term, the sum of the percentage of all sub-categories of GO terms was more than one and the total number of GO terms was bigger than the total number of the unique sequences.

Gene ontology (GO) provided a structured and controlled vocabulary for describing gene products in three categories: molecular functions, cellular components and biological processes, which are helpful to understand the distribution of gene functions of the species at the macro level. We added GO terms using Blast2GO (Conesa et al., 2005), which is based on the automated annotation of all of the unique sequences in the GenBank nr protein database from NCBI. Among the 2824 assembled genes, 1962 were successfully annotated by GO assignments, belonging to one or more sub-categories of GO terms. Among the annotated unigenes, 1813 sequences were assigned to a molecular function category, including catalytic activity (1357 genes), binding (1003 genes), transporter activity (324 genes), nucleic acid binding transcription factor activity (100) and others (Figure 2). Additionally, 980 unigenes are divided into a cellular component category, including cells (759 unigenes), cell parts (759 unigenes), macromolecular complexes (399 unigenes), membrane part (319 unigenes), membranes (407 unigenes), organelles (232 unigenes), and others (Figure 2). In addition, 1722 unigenes are involved in the biological process category, including cellular processes (1297 unigenes), metabolic processes (1399 unigenes), single-organism processes (541 unigenes), establishment of localizations (387 unigenes), localizations (387 unigenes), and others (Figure 2). Because some sequences were assigned to more than one GO term, the total number of GO terms obtained in our dataset was bigger than the total number of the unique sequences.

### COG annotation

**Figure 3:**
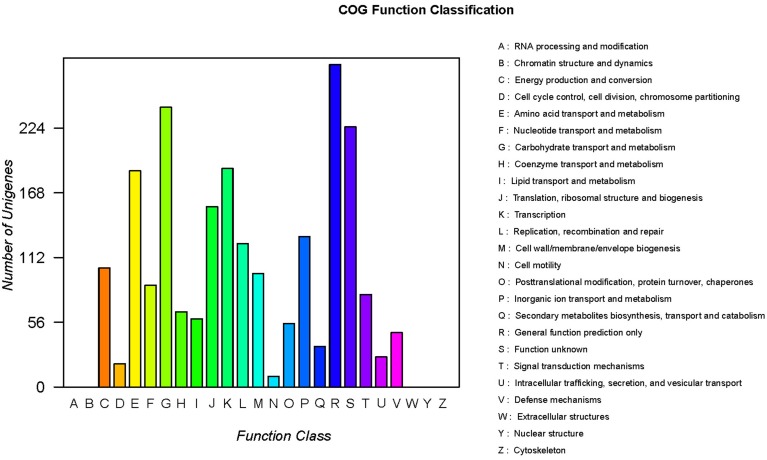
**Clusters of the orthologous group (COG) classifications of the *E. faecalis* sequences**.

COG classification of the unigenes is important for functional annotation and evolutionary studies (Tatusov et al., 2001). Assignments of COG were used to predict and classify the possible functions of the unique sequences. Based on the sequence homology, a total of 2264 DNA-seqs were finally mapped on 20 different COG categories (Figure 3). With the exception of the R or S categories of COG, which are “function unknown” (225 unigenes) or “general function prediction only” (279 unigenes), others were related to the normal physiological metabolism of cells, such as regulation, transport and cell processing. The largest COG group was “carbohydrate transport and metabolism” (242 unigenes), followed by “amino acid transport and metabolism” (187 unigenes), “transcription” (189 unigenes), “translation, ribosomal structure and biogenesis” (156 unigenes), and others (Figure 3). In summary, these terms account for a large fraction of the overall assignments in the *E. faecalis* transcriptome dataset.

### KEGG pathway mapping

The DNAseqs were mapped to the KEGG database to define their metabolic pathways. Pathway-based annotation and analysis helps to further understand the biological function of the genes. According to the KEGG results, 1532 unigenes were classified into 144 KEGG metabolic pathways, representing compound biosynthesis, degradation, utilization and metabolism (Table S1). Many metabolic-related pathways, including fructose and mannose metabolism, pyrimidine metabolism, and fatty acid metabolism, and many biosynthesis pathways, such as the biosynthesis of secondary metabolites, aminoacyl-tRNA biosynthesis, peptidoglycan biosynthesis, fatty acid biosynthesis and so on, were predicted in the KEGG database (Table S1). As in the above GO, one annotated unigene was assigned to one or more of the KEGG pathways.

KEGG pathway analysis and COG analysis are helpful for predicting potential genes and their functions at the whole transcriptome level. The predicted metabolic pathways, together with COG analysis, are useful for further investigations of gene functions in future studies.

### Transcriptome sequences assembly and analysis

Whole genome mRNA sequencing is an attractive method for monitoring global changes in gene expression while overcoming many of the pitfalls of traditional DNA microarrays (Marioni et al., 2008). To identify the genes important for *E. faecalis*-tolerating alkaline stress, we searched for genes that were differentially expressed in pH 10 media. Total RNA was isolated from *E. faecalis* biofilm cells grown for 24-h in TSB or pH 10 media (see the Materials and Methods Section). For the first set of samples, the mRNA was selectively enriched through a single step of rRNA depletion. These samples were fragmented and used to obtain cDNA libraries that were sequenced on an Illumina HiSeq 2000 platform. The detailed sequence information of the transcriptome is listed in Table S2. A total of 1,415,985,842 and 1,810,228,366 bases from 14,350,108 and 18,494,702 Illumina clean reads with a mean read length of 101 bp were obtained from bacterial samples cultured with TSB or pH 10 media, respectively. Of these, 13,910,120 (96.93%) and 18,130,016 (98.03%) clean reads from the control and pH 10 groups were mapped to the reference genome, respectively. Approximately 6,223,986 (43.37%) and 5,288,486 (28.59%) clean reads mapped to CDS.

### Differentially expressed genes

**Figure 4:**
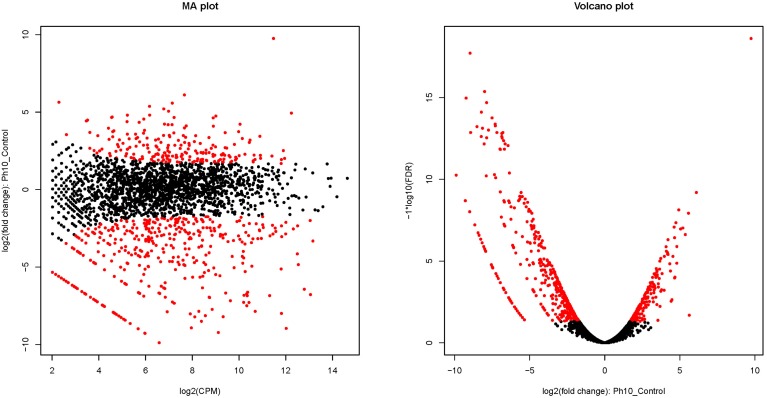
**Differentially expressed genes of *E. faecalis* cultured in pH 10 media**. Red dots indicate differentially expressed genes. Black-colored dots were not considered as significantly differentially expressed. In the figure of the MA plot, the X-axis shows the average count of reads per million reads based on a log_2_ scale, and the Y-axis shows the fold-change values between the control and alkaline group based on a log_2_ scale. In the figure of a volcano plot, the X-axis shows the fold-change values between the control and alkaline groups based on a log_2_ scale, and the Y-axis shows the FDR value of differentially expressed genes based on a -log_10_ scale.

Primary sequence analysis and annotations of all of the unigenes provided us with a great deal of useful information to understand the transcriptome and to further define the DEGs that are induced by alkaline stress. The transcriptome sequencing results revealed 613 genes that were differentially expressed (DEGs) for *E. faecalis* grown in pH 10 medium (Figure 4). Among these DEGs, 211 genes were found to be differentially up-regulated genes (DUGs), and 402 genes were identified as differentially down-regulated genes (DDGs). The data, including the gene ID, gene length, FRKM in the control and pH 10 groups, log2FC1 (pH 10/Control), FDR, up- or down-regulation (pH 10/control) and annotation of all of the DEGs are provided in Table S3.

#### Up-regulated genes (DUGs)

GOG analysis showed the DEGs in 20 predicted pathways. The number of up-regulated genes in each functional category is shown in Table 3. Among the 211 up-regulated genes, the genes encoding amino acid transport and metabolism represented the highest percentage (34 genes; around 16% of all up-regulated genes), while genes related to nucleotide transport and metabolism around 11% (24 genes) of the genes induced by the alkaline stress. Moreover, genes encoding proteins involved in inorganic ion transport and metabolism (16 genes), cell wall/membrane/envelope biogenesis (9 genes), coenzyme transport and metabolism (8 genes), carbohydrate transport and metabolism (7 genes), energy production and conversion (6 genes), and cell cycle control, cell division, chromosome partitioning (2 genes) were up-regulated. In addition, genes classified in the category of genes of unknown function represent a considerable part of the induced genes (26 genes). The majority of up-regulated genes (15 genes) related to amino acid transport and metabolism are genes encoding amino acid or peptide ABC transporter (Table S4). Table S4 gives the full list of genes found to be induced (up-regulated), with fpkm value. Genes predicted to encode hypothetical proteins are excluded from this list.

**Table 3:** **Number of genes that were differentially expressed in alkaline stress**.

**COG category**	**Genes**	**DUGs**	**DDGs**
Energy production and conversion	104	6	24
Cell cycle control, cell division, chromosome partitioning	20	2	0
Amino acid transport and metabolism	187	34	11
Nucleotide transport and metabolism	87	24	1
Carbohydrate transport and metabolism	243	7	41
Coenzyme transport and metabolism	64	8	7
Lipid transport and metabolism	58	1	4
Translation, ribosomal structure and biogenesis	156	8	8
Transcription	193	8	15
Replication, recombination and repair	125	5	10
Cell wall/membrane/envelope biogenesis	96	9	6
Cell motility	9	2	0
Posttranslational modification, protein turnover, chaperones	56	2	10
Inorganic ion transport and metabolism	127	16	12
Secondary metabolites biosynthesis, transport and catabolism	35	1	5
General function prediction only	292	25	25
Function unknown	343	26	62
Signal transduction mechanisms	70	2	6
Intracellular trafficking, secretion, and vesicular transport	27	3	1
Defense mechanisms	47	8	1

#### Down-regulated genes (DDGs)

The numbers of down-regulated genes in each functional category are shown in Table 3. Among the down-regulated genes, those with unknown function are the dominant category (62 genes; 15%), followed by genes involved in carbohydrate transport and metabolism (41 genes; 10%), genes involved in energy production and conversion (24 genes), general function prediction only (25 genes), and genes involved in transcription (15 genes), amino acid transport and metabolism (11 genes), posttranslational modification (10 genes), and translation, ribosomal structure and biogenesis (8 genes). Table S5 gives the full list of genes found to be decreased (down-regulated), with fpkm value. Genes predicted to encode hypothetical proteins are excluded from this list.

### Stress response of *E. faecalis* toward exposure to alkaline stress

Exposure to alkaline repressed universal stress protection genes, including orf02602, orf00603 orf02216, orf02700. The gene (orf01945) encoding Gsp62 and a cold shock protein cspA (orf00260) also showed a significantly decreased expression in alkaline stress. The stress- and starvation inducible *gls24* operon (orf02321-6) was significantly down-regulated. And *gls24*-like gene (orf02786) within the pathogenicity island (PAI) were down-regulated significantly and might possibly contribute to the decrease of *E. faecalis* during growth in pH 10. An organic hydroperoxide resistance gene, *ohr* (Gsp65; orf01411), the NADH peroxidase (orf00385) and the superoxide dismutase gene *sodA* (orf01421) were also down-regulated in alkaline stress. An *ohr* mutant has previously been shown to be less resistant to the oxidative stress generated by 20 mM Tertiary- Butylhydroperoxide, suggesting that *Ohr* may be implicated in oxidative stress resistance in *E. faecalis* (Rincé et al., 2001).

## Discussion

*E. faecalis* are most frequently implicated in human and animal infections (Aakra et al., 2005). It is most commonly isolated from the root canals of teeth with failed endodontic treatment (Sundqvist et al., 1998), which attribute to its resistance to alkaline stress (Bystrom et al., 1985; Evans et al., 2002). Our study also confirmed that *E. faecalis* could survive and form biofilms at pH 10 *in vitro*. Although certain stress response of *E. faecalis* in alkaline media have been studied (Flahaut et al., 1997; Appelbe and Sedgley, 2007), the global gene regulation and interplay during adaptation to alkaline environment remain largely unaddressed. With the present study we provide novel information about the gene regulation relevant to such an adaptation process. The transcriptome sequencing results revealed that 613 genes were differentially expressed (DEGs) for *E. faecalis* grown in pH 10 medium; 211 genes were found to be differentially up-regulated and 402 genes differentially down-regulated.

A comparison of the ATCC33186 genome with the genome features of the published DNA sequence of V583(NC_004668.1), OG1RF(NC_017316.1) and ATCC 29212(NZ_CP008816.1) showed only minor variations: OG1RF consists of 2,739,625 bp with an average G+C content of 37.8%, ATCC 29212 consists of 2,939,973 bp with an average G+C content of 37.5% and V583 consists of 3,218,031 bp with an average G+C content of 37.5%. And ATCC33186 shared 2362, 2284 and 2402 ORFs with V583, OG1RF, and ATCC 29212, respectively, but only shared 3 rRNA genes and 41 tRNA genes. This difference may attribute to only the draft genome of strain ATCC33186 presented in the study, the missing rRNA and tRNA may be much less if the circular chromosomes are completely assembled.

The majority of up-regulated genes (15 genes) related to amino acid transport and metabolism are genes encoding amino acid or peptide ABC transporter. It indicated that the supply and transport of amino acids may be increased in pH 10 media. It has also reported that genes encoding putative amino acid ABC transporter were highly up-regulated in *E. faecalis* exposure to human urine (Vebø et al., 2010). Previous studies on the biosynthetic capacities and nutritional requirements of *E. faecalis* have shown that all strains require histidine, isoleucine, methionine, and tryptophan for growth and that arginine, glutamate, glycine, leucine, or valine was essential for the growth of some strains (Murray et al., 1993; Bourgogne et al., 2008). Moreover, *E. faecalis* is capable of utilizing certain amino acids as energy and carbon sources (Deibel, 1964). Because our data show that the transcription of all genes encoding ABC-transporters were up-regulated, it is possible that *E. faecalis* meets its survival demand by uptaking these essential peptides and amino acids when growing in alkaline stress.

Fourteen genes encoding proteins involved in nucleotide transport and metabolism displayed increased expression under alkaline conditions. The genes for *pyrCDEF* (orf00246, orf00247, orf00248, and orf00252) participated in pyrimidine metabolism, and all of these genes were significantly up-regulated in alkaline stress. The *carAB* (orf00250 and orf00251) operons and *proABC* (orf02358, orf023587, orf00819), which are involved in arginine and proline metabolism, also showed significant up-regulation under alkaline stress. Koo et al. (2011) also reported that the increase of the *carA* expression level had a positive correlation with thymidine production. The repression of the *carAB* operon leads to *Lactobacillus plantarum* FB331 growth inhibition in the presence of arginine (Nicoloff et al., 2004). The significant up-regulation of these genes may promote pyrimidine biosynthetic in the alkaline stress. The capacity for de novo pyrimidine biosynthesis is required for the virulence of some bacteria (Ghim et al., 1999).

The *ade* gene (orf00396) encoding adenine deaminase and *add* gene (orf01977) encoding adenosine deaminase were markedly up-regulated in alkaline stress. Adenine deaminase (ADE) catalyzes the conversion of adenine to hypoxanthine and ammonia (Kamat et al., 2011). The transcription of *guaD* (orf00812) encoding guanine deaminase, *guaC* (orf00814) encoding guanosine monophosphate (GMP) reductase and *guaB* (orf02514) encoding inosine 5′-monophosphate dehydrogenase (IMPDH) were markedly enhanced in alkaline stress. IMPDH catalyzes the pivotal step in guanine nucleotide biosynthesis (Mandapati et al., 2014). GMP reductase catalyzes the reductive deamination of GMP to inosine monophosphate (IMP). GMP first binds to the enzyme, followed by NADPH binding, and NADP(+) dissociates first, followed by IMP release. GMP reductase also plays an important role in the conversion of the nucleoside and nucleotide derivatives of guanine to adenine nucleotides. In addition, as a member of the purine salvage pathway, it plays a critical role in reutilization of free intracellular bases (Li et al., 2006; Martinelli et al., 2011). Additionally, the *gmk* gene (orf01089) encoding guanylate kinase was also up-regulated. Guanylate kinase is involved in the reversible transfer of phosphate groups from ATP to GMP. GMP and ATP serve as the most effective phosphate acceptors and donor, respectively (Gupta et al., 2014). This means nucleotide metabolism pathway is enhanced in alkaline stress.

The majority proteins of down-regulated genes involved in energy production and conversion encoded are related to ATP synthase. The transcript level of the *atpE* gene (orf00031) and *atpABCDFG* (orf01626-33), encoding F0F1 ATP synthase subunits, were significantly down-regulated in pH 10 media. ATP hydrolysis relies on FoF1-ATPase, which in *E. faecalis*, is composed of the Fo membrane proton channel and the F1 cytoplasmic catalytic site (Kakinuma, 1998; Aslangul et al., 2006). The AtpE protein is a V/A-type H+-transporting ATPase subunit E, and the H+-ATPase functions to pump protons out of the cell to form an electrochemical proton gradient (Heefner, 1982). Additionally, the *nhaC* gene (orf01548), encoding a Na+/H+ antiporter was significantly down-regulated in pH 10 media. According to Kakinuma and Igarashi (1990), the proton motive force is dramatically decreased and the activity of the H^+^-ATPase is very low at pHs above 8, and at pH 10, the proton potential proceeds to zero. Under these conditions, the Na^+^/H^+^ antiporter, which relies on H^+−^ATPase, does not operate. Therefore, the transcription of the genes decreased, causing an impairment of cytoplasmic alkalization activity, which can create conditions for cells survival in alkaline stress.

Most DEGs encoding proteins involved in carbohydrate transport and metabolism displayed decreased expression under alkaline conditions. 15 genes *celABC* (orf01842, orf02475, orf02476, orf02813), *manXYZ* (orf01871-4, orf01078-81) and *scrA* (orf00808), *bglF* (orf01289) involved in encoding the IIABCD component of the phosphotransferase system (PTS) were significantly down-regulated under alkaline stress. These include the predicted lactose/galactose and gluconate PTS systems, the latter is part of a predicted metabolic pathway that facilitates gluconate uptake and catabolism via the mannonate route (Deutscher et al., 2006). This is consistent with a recent investigation that showed that genes encoding PTS were down-regulated in other stress conditions (Vebø et al., 2010). PTS is a distinct method used by bacteria for sugar uptake where the source of energy is phosphoenolpyruvate (PEP). It is involved in transporting many sugars into bacteria, including glucose, mannose, fructose, and cellobiose. PTS sugars can differ between bacterial groups, with each group evolving to mirror the most suitable carbon sources available in the environment. This implies that substrates besides glucose might play a role for growth of *E. faecalis* in alkaline stress. Moreover, *gnd* (orf01075) encoding 6-phosphogluconate dehydrogenase-like protein, *uxuA* (orf01082) encoding mannonate dehydratase, *bglA* (orf01290-1, orf01293) encoding 6-phospho-beta-glucosidase, *gpmB* (orf01292) encoding the phosphoglycerate mutase family protein and *fruK* (orf01488) encoding 1-phosphofructokinase were down-regulated in alkaline stress. This suggested that the glycolytic pathway of *E. faecalis* becomes slack during growth in alkalis; the cells initiate use of less preferred carbon and energy sources, especially fructose and mannose. The operons orf00241, orf01641, orf01875, and orf02474, encoding glycosyl hydrolase family proteins, were all down-regulated, implying that glycan degradation may also be decreased.

*E. faecalis* is equipped with two pathways for glycerol dissimilation. Glycerol can either be phosphorylated first by glycerol kinase and then oxidized by glycerol-3-phosphate oxidase (the glpK pathway) or first be oxidized by glycerol dehydrogenase and then phosphorylated by dihydroxyacetone kinase (the dhaK pathway) (Bizzini et al., 2010). In our study, the *GlpF* operon (orf00557) encoding glycerol uptake facilitator protein, *glxK (orf01600)* encoding glycerate kinase and *dhaKL* (orf02382-3) encoding dihydroxyacetone kinase were significantly down-regulated, suggesting that glycerol metabolism was reduced. The enzymes of glycerol metabolism are crucial for the pathogenicity of *M. pneumoniae* but also for other essential, yet to be identified, functions in the *M. pneumoniae* cell (Hames et al., 2009). The down-regulation of *Glp* and *glxK* may influence other functions or the pathogenicity of *E. faecalis*.

However, the *metE* gene (orf01358) encoding methionine synthase was significantly down-regulated under alkaline stress. It has been demonstrated that the stabilized MetE enzymes alone were responsible for the accelerated growth and higher survival observed in *E. coli* cells at elevated temperatures (Mordukhova and Pan, 2013). This down-regulation may reduce the synthesis of cysteine and methionine, so cells grow slower. Orf01979-encoding lipase/acylhydrolase and orf02404-encoding peptidase T, which cleaves the N-terminal amino acid of tripeptides, were also down-regulated in alkaline stress. The hydrolytic enzymes lipase and peptidase were found to be involved in the life cycle of *Enterococcus* species (Fisher and Phillips, 2009). This may also explains the slower biofilm growth in alkaline stress.

In our study, *E. faecalis* down-regulated stress protection genes in response to alkaline stress. Inactivation of *gls24* and *glsB* (orf02322 and orf02323, respectively) has been reported to have a pleiotrophic effect on cell morphology and stress tolerance in *E. faecalis* (Giard et al., 2000). In our previous study, we also have observed cells under alkaline stress developed irregular shapes with pits on the cell wall surface that resemble separation anomalies (Ran et al., 2013). However, *E. faecalis* induces *gls24 gene* and some other stress protection genes in response to other different stress conditions (Giard et al., 1997; Vebø et al., 2009, 2010). Proteomic analyses with systematic exposure to various stresses have previously identified six genes encoding general stress response proteins (GSPs) which were up-regulated in *E. faecalis* by a wide variety of environmental stimuli (Rince et al., 2000). The reasons for the contrary results may be that the time of *E. faecalis* exposure to the stress conditions does not exceed 60 min in these studies, but in our study *E. faecalis* grown in alkaline stress for 24 h before analysis, the survival cells had adapted to the alkaline environment, so most of the differently regulated stress response genes were down-regulated which indicated the sensitivity to alkaline stress might decrease and its stress tolerance in alkaline stress might increase.

## Conclusion

In this study, de novo transcriptome sequencing of *E. faecalis* using the Illumina 2000 was performed for the first time. A total of 18,494,702 high-quality transcriptome reads were obtained, giving rise to an average of 101 bp per read. Compared to the control group, many genes involved in carbohydrate transport and metabolism and energy production and conversion were significantly down-regulated, but genes involved nucleotide and amino acid transport and metabolism were significantly up-regulated, which might contribute to adaptation and survival of *E. faecalis* in alkaline stress. Most interesting is mostly differently expressed stress response genes were down-regulated in alkaline stress. This study provides new insights into the adaptive process of *E. faecalis* to growth and persistence in alkaline stress, and represent a first step in the process to identify proteins that might be important to the survival of *E. faecalis* in high pH environments. It is hoped that future studies can incorporate the rapid advances underway in metabolomics and proteomics. Extrapolation of the present data to the clinical situation can only be speculative. However, the present data may contribute toward better understanding the survival strategies of *E. faecalis* following prolonged exposure to pH levels similar to those encountered in root canals receiving intracanal Ca(OH)_2_ treatment.

## Nucleotide sequence accession number

The draft genome sequence of *E. faecalis* ATCC33186 and the RNA-seq sequencing data have been deposited in NCBI database under accession number SRP056562 (DNA sequence) and SRP056563 (RNA sequence).

### Conflict of interest statement

The authors declare that the research was conducted in the absence of any commercial or financial relationships that could be construed as a potential conflict of interest.

## Supplementary material

Table S1 **KEGG summary of *E. faecalis* sequences (XLS)**.

Table S2 **The detail sequence information of the transcriptome**.

Table S3 **The data of all of the gene transcription information (XLS)**.

Table S4 **The list of genes found to be induced (up-regulated) in alkaline stress**.

Table S5 **The list of genes found to be decreased (down-regulated) in alkaline stress**.
