# Supplementary material for: Transcriptome analysis of Enterococcus faecalis in response to alkaline stress
Source: Front Microbiol. 2015 Aug 7;6:795. doi: 10.3389/fmicb.2015.00795 (PMC4528170; doi:10.3389/fmicb.2015.00795)
Supplement: Table S2 — The detail sequence information of the transcriptome. [file Table2.DOCX]

Table S2 The detail sequence information of the transcriptome

| Dataset name | control | pH 10 |
| --- | --- | --- |
| Total raw reads | 14,575,418 | 18,878,650 |
| Total clean reads | 14,350,108 | 18,494,702 |
| Q20 percentage | 99.08 | 98.8 |
| N percentage | 0 | 0 |
| GC percentage | 39.99 | 38.11 |
| Clean reads mapped to  genome (%) | 96.93% | 98.03% |
| Clean reads mapped to  CDS (%) | 43.37% | 28.59% |
| Clean reads mapped to  rRNA (%) | 0.40% | 0.15% |
