# Supplementary material for: Transcriptome analysis of Enterococcus faecalis in response to alkaline stress
Source: Front Microbiol. 2015 Aug 7;6:795. doi: 10.3389/fmicb.2015.00795 (PMC4528170; doi:10.3389/fmicb.2015.00795)
Supplement: Supplementary file 6 [file DataSheet1.DOCX]

**Q7: Thank you. It is now perfectly clear.**

**Just in case, do you have any real-time PCR results to show?**

**Answer:** Yes, we can show the real-time PCR results. Five genes involved in stress response and cell division were randomly selected. Primers were listed as follows:

16S-Eub338 ACTCCTACGGGAGGCAGCAG

16S-Eub518 ATTACCGCGGCTGCTGG

dnaK-F CAGTTAACCCTGACGAAG

dnaK-R TGACAACGGTGTTACGTC

groEL-F CTGTTTCAGTTGCAGCAC

groEL-R CAAATCGGCGAAACAACG

gls24-F TAACAGTCGATGGCGGCTTT

gls24-R CAGCGACTTGTTTTTTACCAACTTC

ftsZ-F CCGTCAAACAAGACAAGCGG

ftsZ-R TCCCAATCGCCAAAAGCACT

salB-F gcaccagttgcctcttcttc

salB-R cagttgtttgttgcgtgctt

And the result are shown as follows:

1. The amplification curves and melting curves of 16s


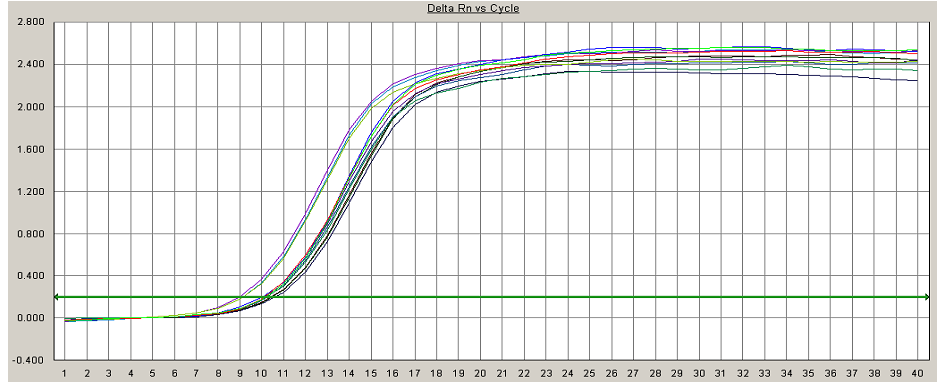

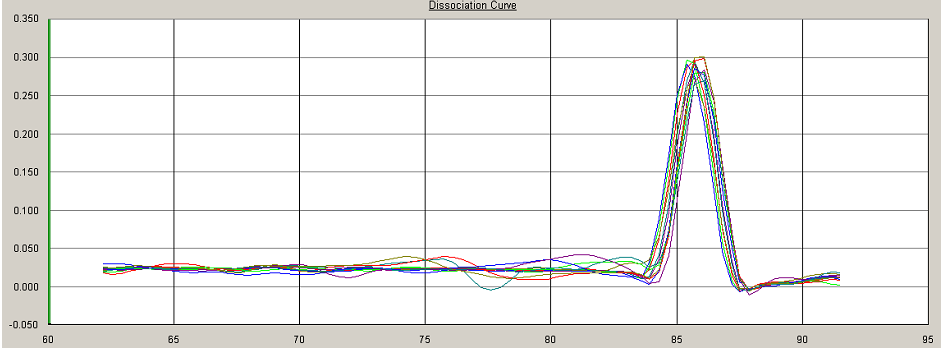


1. The amplification curves and melting curves of dnaK


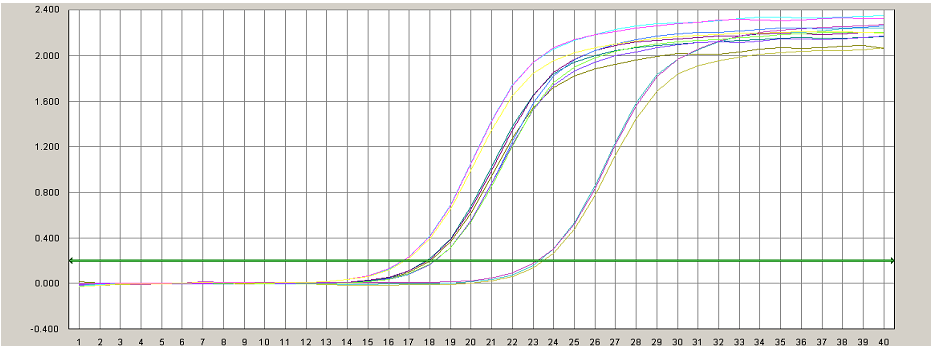


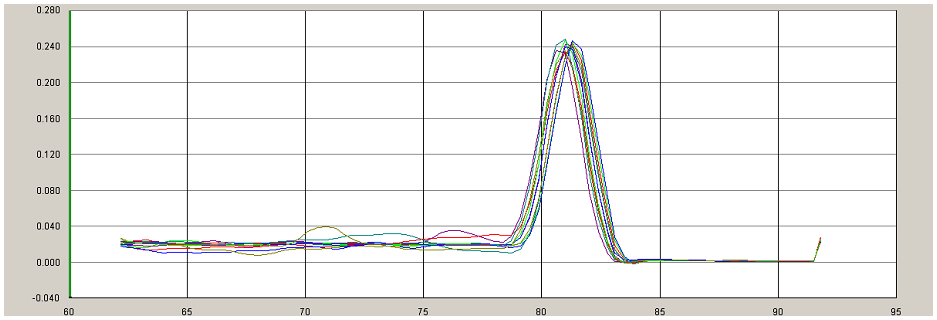


3. The amplification curves and melting curves of groEL


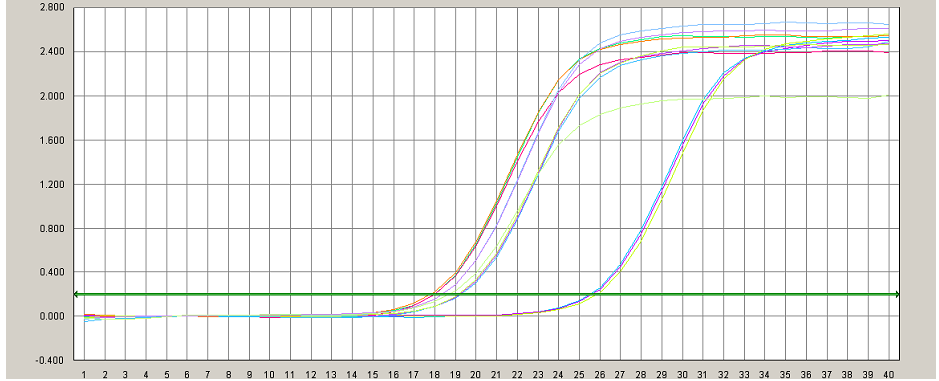


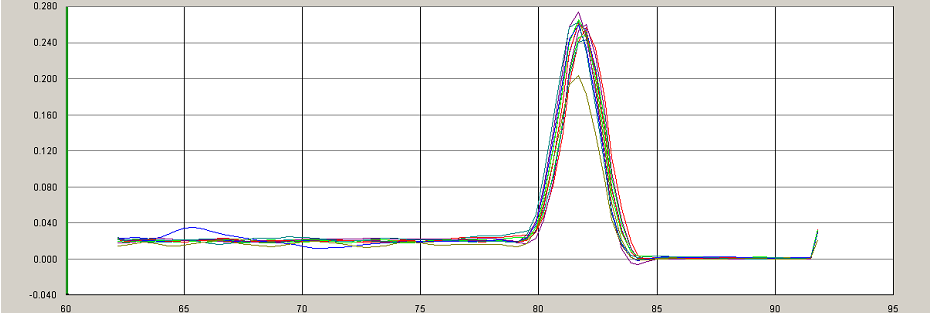


4. The amplification curves and melting curves of gls24


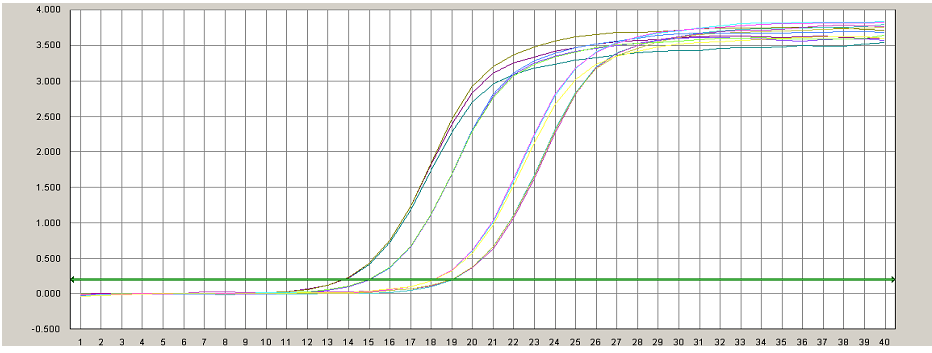


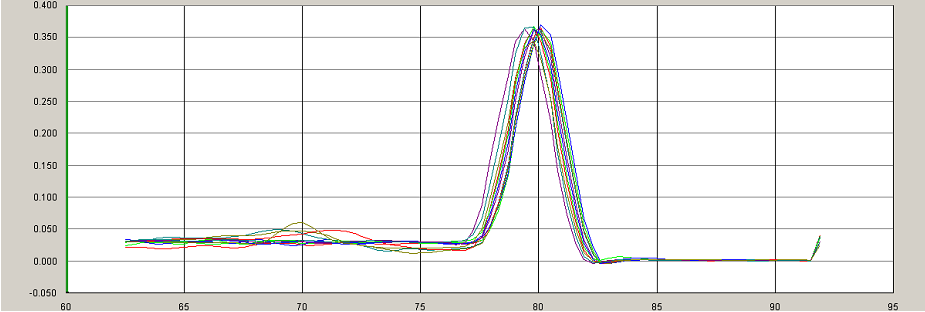


5. The amplification curves and melting curves of ftsZ


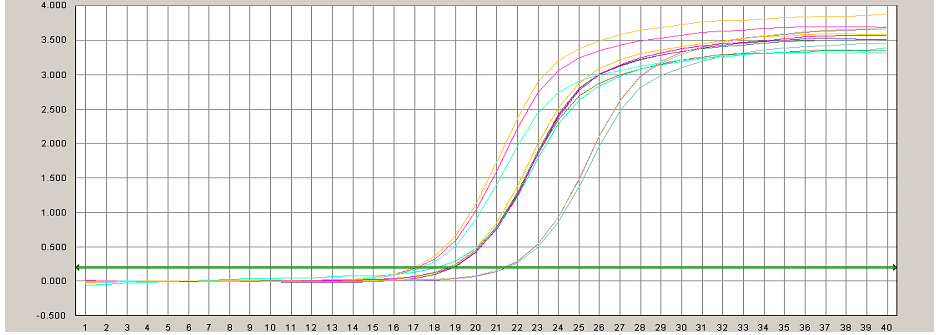


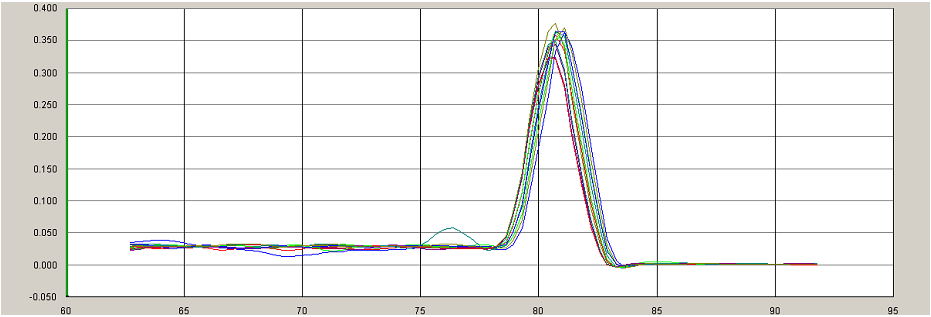


6. The amplification curves and melting curves of salB


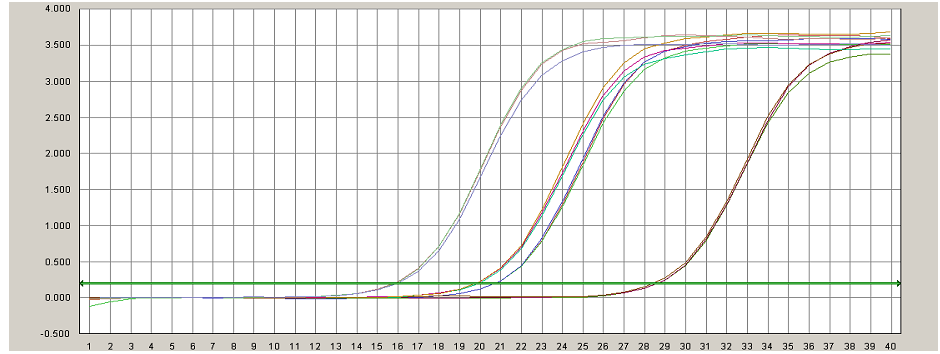


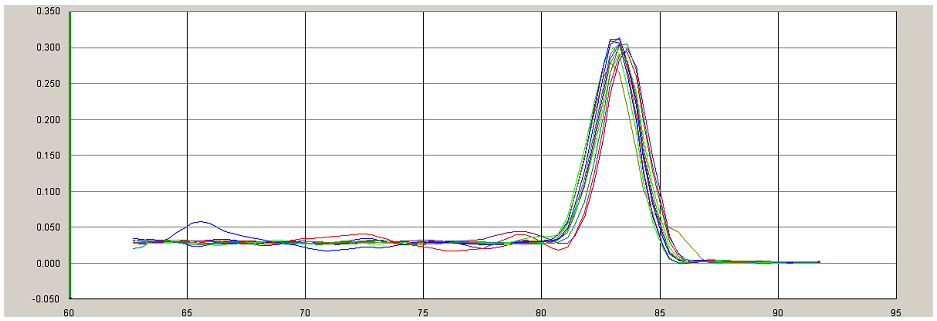


7. Table A:The fold change of these genes by real time PCR and sequencing

| gene name | genes | CT value(mean) | | | | | | 2 ^-(∆∆Ct)^ | log2FC1(Ph10/Control)by sequence |
| --- | --- | --- | --- | --- | --- | --- | --- | --- | --- |
|  |  | 16S of control | Interest gene of control | 16S of pH 10 | | Interest gene of pH 10 | |  |  |
| salB | orf02232 | 10.51 | 20.76 | | 10.14 | 15.92 | | 22.2 | 3.43 |
| ftsZ | orf01273 | 10.51 | 18.78 | | 10.14 | 17.19 | | 2.33 | 2.17 |
| dnaK | orf00476 | 10.51 | 17.92 | | 10.14 | 19.26 | | -3.2 | -2.0 |
| groEL | orf01612 | 10.51 | 17.94 | | 10.14 | 18.35 | | -1.7 | -2.54 |
| gls24 | orf02322 | 10.51 | 13.80 | | 10.14 | 16.06 | -6.3 | | -6.43 |

PS: The real-time PCR and sequence were conducted with another two stress conditions, but the amplification curves and melting curves can’t be shown separately, so there were 12 amplification curves and melting curves (four test groups and each group three duplication ) for every gene.
